# Anti-HMGB1 auto-Abs influence fatigue in patients with Crohn’s disease

**DOI:** 10.1177/17534259211014252

**Published:** 2021-05-03

**Authors:** Ingeborg Kvivik, Tore Grimstad, Grete Jonsson, Jan T. Kvaløy, Roald Omdal

**Affiliations:** 1 Research Department, Stavanger University Hospital, Norway; 2 Unit of Gastroenterology, Department of Internal Medicine, Stavanger University Hospital, Norway; 3 Department of Clinical Science, Faculty of Medicine, University of Bergen, Norway; 4 Department of Medical Biochemistry, Stavanger University Hospital, Norway; 5 Department of Chemistry, Bioscience and Environmental Engineering, University of Stavanger, Norway; 6 Department of Mathematics and Physics, University of Stavanger, Norway; 7 Clinical Immunology Unit, Department of Internal Medicine, Stavanger University Hospital, Norway

**Keywords:** Anti-HMGB1 antibodies, Crohn’s disease, fatigue, HMGB1, sickness behaviour

## Abstract

Fatigue is common in all chronic inflammatory and autoimmune diseases. A conceptual model for understanding the biological basis of fatigue describes it as being a part of the sickness behaviour response generated by pro-inflammatory cytokines and other mediators. We hypothesised that the pro-inflammatory high mobility group box 1 (HMGB1) protein is a fatigue-inducing molecule and that auto-Abs against HMGB1 reduce fatigue. We measured Abs against disulphide (ds) HMGB1 and fully reduced (fr) HMGB1 in plasma from 57 patients with Crohn’s disease. Fatigue was rated using the fatigue visual analogue scale (fVAS) and disease activity with faecal calprotectin, C-reactive protein and the Simple Endoscopic Score for Crohn’s disease. Multivariable regression models identified anti-dsHMGB1 and anti-frHMGB1 Abs as the strongest contributing factors for fVAS scores (*B* = −29.10 (*P* = 0.01), *R*^2^ = 0.17, and *B* = −17.77 (*P* = 0.01), *R*^2^ = 0.17, respectively). Results indicate that anti-HMGB1 auto-Abs alleviate fatigue possibly by down-regulating HMGB1-induced sickness behaviour.

## Introduction

High mobility group box 1 (HMGB1) is a non-histone chromatin-binding protein abundantly found in all cell nuclei and is composed of 215 aa with two DNA-binding domains (A and B boxes) as well as an acidic, negatively charged C-terminal.^1^ This evolutionarily well-conserved protein^2^ serves fundamental and divergent tasks both intracellularly and extracellularly. In the nuclei of resting cells, HMGB1 regulates DNA transcription and organisation of DNA and nucleosomes, but in this state, it has no known immune function.^3^ Cell stress, cellular injury or pathogens may stimulate HMGB1 translocation from the nucleus to the cytoplasm and subsequent release into the extracellular space.^4^

HMGB1 contains three conserved redox-sensitive cysteines, and the bioactivity of HMGB1 is dependent on modifications of these cysteine residues. The protein plays a key role in the clearance of various bacterial and viral infections and enhances rapid clearing of intracellular pathogens and endogenous damage-associated molecular patterns (DAMPs).

When macrophages and monocytes directly encounter pathogens or DAMPs, HMGB1 is actively secreted into extracellular space by the immune cells. The protein activates TLR4 and elicits strong activation of innate immune cells and inflammatory responses.^5–7^

Many clinical studies have described the central role of HMGB1 in inflammation, and recent studies have also pointed out its potential as a therapeutic target.^8^ Increased blood concentrations of HMGB1 are observed during infections and in chronic inflammatory and autoimmune diseases.^9^ In patients with systemic lupus erythematosus (SLE), blood concentrations of HMGB1 correlate with disease activity.^10^

In patients with Crohn’s disease, a chronic inflammatory bowel disease (IBD), the blood concentration of HMGB1 is higher than that in healthy subjects.^11^ Inflamed cells in the intestine release cytokines and DAMPs, and HMGB1 can also be detected in the faeces.^12^,^13^ Further evidence for the pro-inflammatory actions of HMGB1 in IBD is derived from the finding that inhibition of HMGB1 by neutralising anti-HMGB1 Abs resulted in reduced inflammation in an animal model of colitis and sepsis.^14^ The potential for reversing and preventing the activation of innate immunity by targeting HMGB1 in diverse models of sterile and infection-induced conditions has been reviewed by Andersson and Tracey.^4^

HMGB1 may act as an auto-Ag and induce the production of anti-HMGB1 auto-Abs. These auto-Abs are detected in a large proportion of patients with autoimmune diseases as well as healthy humans.^15^ The production of Abs against highly conserved endogenous proteins is rather unusual, and its potential role is not fully understood.^16^ In SLE patients, Ab levels appear to correlate with the SLE Disease Activity Index (SLEDAI) and coincide with renal involvement.^17^,^18^ Interestingly, a study by Barnay-Verdier et al. indicated that anti-HMGB1 Abs had protective functions against septic shock and thus may represent an Ab-mediated down-regulation of the immune response in autoimmune diseases.^19^ The ability of extracellular HMGB1 to induce the production of its own neutralising Abs suggests an inherited negative feedback loop that limits the level of innate immune activation.

Chronic fatigue, a condition characterised by ‘an overwhelming sense of tiredness, lack of energy, and feeling of exhaustion’, is common in all chronic inflammatory diseases.^20^ The biological definition of fatigue is that fatigue is a part of sickness behaviour – a response observed in animals during infections. Sickness behaviour is generated in the brain when IL-1β binds to specific IL-1 receptors on neurons. IL-1β can pass from the periphery to the CNS across the blood–brain barrier, or it can be produced in the brain by activated microglia.^21^,^22^ Disulphide (ds) HMGB1 and other DAMPs are among the molecules that bind to TLR4 on macrophages and microglia and stimulate proinflammatory cytokine production, thus inducing sickness behaviour.^7^,^23^ TLR4-blocking peptides are able to prevent this behaviour in animal studies.^24^ Another route for IL-1β production is through fully reduced (fr) HMGB1, which binds to receptor for advanced glycation end products (RAGE) on macrophages and microglia and induces the release of pro-inflammatory cytokines.

We hypothesise that anti-HMGB1 Abs down-regulate HMGB1-driven immune activity leading to lower IL-1β-driven macrophage and microglia activation and thus lower fatigue. We measured Abs in blood against the redox variants dsHMGB1 and frHMGB1 and revealed a strong association with fatigue severity.

## Patients and methods

### Study participants

Fifty-seven patients with newly diagnosed and untreated Crohn’s disease were consecutively recruited from the Department of Gastroenterology at Stavanger University Hospital. In addition, 28 healthy control subjects among the hospital’s staff and their friends and family members with no history of immunological or neurological disease were recruited (Table 1). All subjects were clinically examined, and they filled out the fatigue visual analogue scale (fVAS) questionnaire under supervision and guidance. The fVAS is a 100-mm horizontal line with vertical anchors. The wording on the left end (0 mm) is ‘No fatigue’ and on the right end (100 mm) is ‘Fatigue as bad as it can be’. It is a frequently used and widely accepted generic and unidimensional fatigue instrument for rating fatigue in many diseases.^25^

**Table 1.** Demographic and laboratory variables in 57 patients with newly diagnosed untreated Crohn’s disease and 28 healthy subjects.

	Patients with Crohn’s disease (*n*=57)	Healthy control subjects (*n*=28)	*P* Value
Age, yr	31 (16–78)	43 (23–66)	0.002
Male/female, *n* (%)	22 (38.6)/35 (61.4)	5 (17.9)/23 (82.1)	0.08
fVAS, scores (*n* = 56)	54.5 (1–98)	14 (2–56)	< 0.001
Hb, g/l, *M* (*SD*)	12.3 (1.8)		
CRP, mg/dl	8.4 (1.0–139)		
F-calprotectin, mg/kg (*n* = 52)	338 (15–4432)		
SES-CD	8 (1–37)		
Disease distribution (*n* = 56), *n* (%)			
Ileum	24 (42.9)		
Colon	8 (14.3)		
Ileocolon	24 (42.9)		
Anti-dsHMGB1 Abs^a^	0.17 (0.04–1.32)	0.18 (0.04–1.41)	0.43
Anti-frHMGB1 Abs^a^	0.04 (0.04–1.18)	0.04 (0.04–1.05)	0.58

Medians and ranges are given unless otherwise stated.

^a^Absorbance at 490 nm.

dsHMGB1: disulphide high mobility group box 1 protein; f-calprotectin: faecal calprotectin; frHMGB1: fully reduced HMGB1; fVAS: fatigue visual analogue scale; SES-CD: Simple Endoscopic Score for Crohn’s Disease.

### Disease activity measures

Disease activity in patients was assessed by C-reactive protein (CRP) levels in blood, faecal (f-) calprotectin levels, and by colonoscopic evaluation using the Simple Endoscopic Score for Crohn’s disease (SES-CD).^26^

### Laboratory tests

Routine laboratory tests were performed, including tests for haemoglobin, white blood cell count and thrombocytes. In addition, blood was drawn into EDTA tubes, cooled on ice and centrifuged at 2800 *g* for 15 min at 4°C. Plasma was aliquoted and stored at −80°C until further analysis.

### ELISA for anti-HMGB1 auto-Abs

An in-house ELISA method for detection of Abs in plasma against different redox isoforms of HMGB1 was performed as follows. Each Greiner Bio-One (Frickenhausen, Germany) medium 96-well plate was divided into three sections and coated overnight (4°C) with 50 µl dsHMGB1 (2 µg/ml), 50 µl frHMGB1 (2 µg/ml) (HMGBiotech, Milan, Italy) in PBS (Thermo Fisher Scientific, Rockford, IL) or pure PBS. After coating, the wells were blocked for 1 h with 300 µl 20% FBS (GIBCO, Thermo Fisher Scientific) in PBS w/0.05% Tween-20 (VWR Chemicals, Fontenay-sous-Bois, France). Each plasma sample was diluted 1:25 in blocking solution before 50 µl of it was added in triplicate to each of the three coatings and incubated for 2 h. Blocking solution was used as a solvent blank, and two different plasma samples were analysed on all plates to determine the between-plate variation. Secondary Ab (anti-polyvalent immunoglobulin (IgG, A, M) peroxidase conjugate (A8400), 0.9 mg/ml; Sigma–Aldrich, St Louis, MO) diluted 1:1000 in blocking buffer was added (100 µl/well) for 1 h incubation. The wells were washed, and bound Ab was detected by adding 100 µl OPD-solution (OPD-tablets (#34006) and peroxide substrate buffer (#34062); Thermo Fisher Scientific). The reaction was stopped with 100 µl 2 M H_2_SO_4_ (VWR Chemicals) after 30 min. Incubations were performed at room temperature, and each plate wash was repeated five times using 400 µl PBS w/0.05% Tween-20. Absorbance was measured at 490 nm (Gen5 plate reader; BioTek Instruments, Winooski, VT).

Absorbance for non-specific binding measured in PBS-coated wells was subtracted from the absorbance measured in the Ag-coated wells. The remaining absorbance is a relative measure of the Ab level in each sample. The limit of detection (LOD) for anti-dsHMGB1 and anti-frHMGB1 Abs was calculated from the mean absorbance+2.5 *SD* of the solvent blank. Sample absorbance below the LOD was set to LOD/√2 for calculations. Between-plate variation was assessed by comparing the mean absorbance of the internal quality controls and found to be 15.3% and 17.8% for anti-dsHMGB1 and anti-frHMGB1 Abs, respectively.

### Statistical analysis

Normal distribution of the data was tested by using the Shapiro–Wilk test. Demographic and clinical data are reported as the mean ± SD if normally distributed, otherwise as median (range). Means of normally distributed data in patients versus those in healthy control subjects were compared using a simple *t*-test, while non-normally distributed data were compared by using the Mann–Whitney *U*-test. The differences in categorical variables between the groups were analysed by using the chi-square test.

When developing regression models, univariable linear regression analyses were first performed using fVAS scores as the dependent variable and age, sex, Hb, CRP, f-calprotectin, SES-CD and the two different anti-HMGB1 Abs as independent variables. Independent variables with *P* < 0.2 (CRP, f-calprotectin, SES-CD, anti-dsHMGB1 and anti-frHMGB1) in addition to age and sex were then selected into the multivariable regression models. As the anti-HMGB1 Ab variants showed high levels of intercorrelation, we fitted two separate multivariable models: anti-dsHMGB1 Abs and anti-frHMGB1 Abs, respectively. Finally, backward stepwise selection models were fitted to exclude non-significant independent variables.

*P*-values < 0.05 were considered significant. The analyses were performed using IBM SPSS Statistics for Windows v26 (IBM Corp., Armonk, NY), and the figures were made in RStudio 1.1.463 with R v3.5.1 (The R Foundation for Statistical Computing, Vienna, Austria).

### Ethical considerations

This study was approved by the Norwegian Regional Ethics Committee (REK 2011/2631) and carried out in compliance with the principles outlined in the Declaration of Helsinki. All patients provided written informed consent to participate in the study. The study was registered at ClinicalTrials.gov (NCT01551563).

## Results

### Baseline characteristics

Selected demographic and laboratory data are presented in Table 1. Patients were younger than the healthy subjects and had higher fVAS scores. There were no significant differences in the absorbance levels of either anti-dsHMGB1 or anti-frHMGB1 Abs between patients and healthy control subjects.

### Fatigue and disease activity markers

No statistically significant associations occurred between disease activity markers (SES-CD, f-calprotectin and CRP) and fVAS scores in the univariable or multivariable linear regression models (Tables 2 and 3).

**Table 2.** Associations between fatigue (fVAS scores) and selected demographic and laboratory variables in patients with Crohn’s disease.

	fVAS (*n* = 56)		
	*B*	*R* ^2^	*P* Value
Age, yr	−0.24	0.02	0.27
Sex	8.93	0.03	0.20
Hb, g/l	−1.61	0.01	0.41
CRP, mg/dl	0.17	0.05	0.09
F-calprotectin, mg/kg (*n* = 52)	0.01	0.04	0.15
SES-CD	0.63	0.04	0.13
Anti-dsHMGB1 Abs^a^	−30.99	0.14	0.005
Anti-frHMGB1 Abs^a^	−37.71	0.17	0.001

Univariable regression with fVAS as the dependent variable.

^a^Absorbance at 490 nm.

**Table 3.** Multivariable regression models including age and sex to show the effect of anti-HMGB1 Abs on fatigue severity.

	fVAS (*N* = 56)	
	*B*	*P*-value
Anti-dsHMGB1 Abs^a^	−27.17	0.03
Age, yr	−0.28	0.26
Sex	6.35	0.40
CRP, mg/dl	0.04	0.77
F-calprotectin, mg/kg (*n* = 52)	0.002	0.63
SES-CD	0.45	0.46
*Model summary*	*R*^2^ = 0.23, *P* = 0.07	
Anti-frHMGB1 Abs^a^	−15.40	0.04
Age, yr	−0.26	0.29
Sex	8.25	0.26
CRP, mg/dl	0.03	0.81
F-calprotectin, mg/kg (*n* = 52)	0.001	0.83
SES-CD	0.47	0.44
*Model summary*	*R*^2^ = 0.21, *P* = 0.09	

fVAS scores as the dependent variable. Multivariable regression model before backward selection.

^a^Absorbance at 490 nm.

### Fatigue and anti-HMGB1 Abs

**Figure 1.**
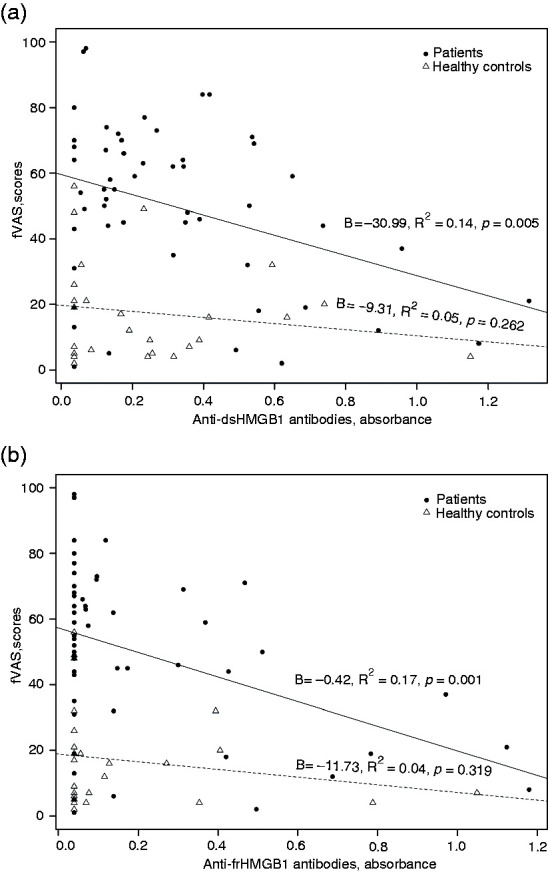
Association between fatigue and levels of anti-HMGB1 Ab variants. Univariable linear regression analysis in 56 patients with Crohn’s disease and 28 healthy control subjects, using the fatigue visual analogue scale (fVAS) as the dependent variable and anti-dsHMGB1 Abs (a) or anti-frHMGB1 Abs (b) as the independent variable. For each group, a fitted regression line is shown, continuous for patients and dotted for healthy control subjects.

In univariable regression analysis, the increasing concentrations of Abs against both isotypes of HMGB1 were inversely associated with fVAS scores in the patient group, whereas no associations were found for healthy control subjects (Table 2 and Figure 1a and b).

We performed multivariable regression analysis with fVAS scores as the dependent variable and anti-dsHMGB1 or anti-frHMGB1 Abs among the disease activity independent variables (CRP, f-calprotectin and SES-CD), age and sex. This yielded *R*^2^=0.23, *P* = 0.07, and *R*^2^ = 0.21, *P* = 0.09, respectively, for the two models (Table 3). Anti-HMGB1 ab levels for both variants were strongly and inversely associated with fVAS scores. Applying backward stepwise selection while keeping the variables age and sex in the model, anti-HMGB1 Abs were identified as the most contributing factor for fVAS scores, yielding *B* = −29.10, *P* = 0.01, *R*^2^ = 0.17 for anti-dsHMGB1 Abs, and *B* = −17.77, *P* = 0.01, *R*^2^ = 0.17 for anti-frHMGB1 Abs.

## Discussion

The main finding of this study is that high levels of anti-HMGB1 auto-Abs in blood are associated with less fatigue in patients with Crohn’s disease. This observation supports the hypothesis that in inflammatory conditions, fatigue, at least to some extent, is generated by pro-inflammatory cytokines and that HMGB1 is involved in fatigue signalling pathways and boosts this mechanism. Of the various known pro-inflammatory cytokines, IL-1β has been shown to be a central actor in fatigue generation. In the brain, this molecule binds to a complex of the IL-1 receptor type 1 (IL-1RI) and a brain subtype of the accessory protein (AcP), the AcPb.^27^,^28^ This leads to neuronal activation, which in turn induces sickness behaviour, of which fatigue is a major component.

**Figure 2.**
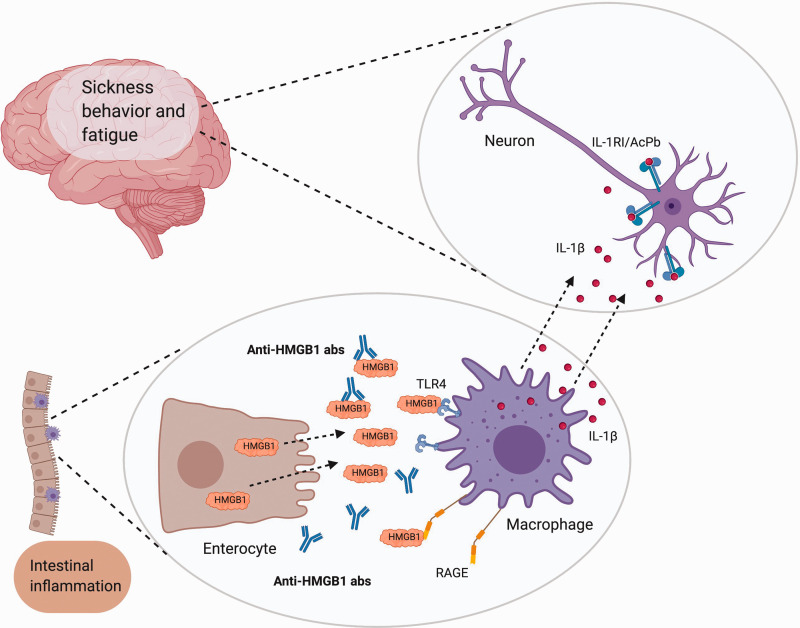
A model for HMGB1-induced fatigue and sickness behaviour. Inflamed and damaged cells in the intestine release HMGB1 protein. HMGB1 activate macrophages by binding TLR4 and receptor for advanced glycation end-products (RAGE). This leads to release of pro-inflammatory cytokines, including IL-1β. In the central nervous system, IL-1β binds to a complex of IL-1 type I (IL-1RI) receptors and accessory protein (AcPb) on neuronal cells and induces sickness behaviour and fatigue. Anti-HMGB1 Abs bind HMGB1 and thus down-regulate HMGB1-induced fatigue. Created with BioRender.com.

Therapeutic blockade of IL-1 alleviates fatigue in patients with rheumatoid arthritis, diabetes type 2, primary Sjögren’s syndrome and cancer-related fatigue.^29–32^ HMGB1 activates macrophages and microglia to produce and release IL-1β. When anti-HMGB1 Abs are present, lower amounts of HMGB1 is available for stimulation of macrophages and microglia. Thus, lower production and release of IL-1β and other pro-inflammatory cytokines follows, and lower neuronal activation and sickness behaviour/fatigue is observed. HMGB1 and anti-HMGB1 Abs therefore add to the network of molecules that influence fatigue (Figure 2).^33^

To the best of our knowledge, this is the first study to investigate the association between fatigue and HMGB1 in humans. A few animal studies support the fatigue-inducing effect of HMGB1. In a study by Frank et al., administering dsHMGB1 directly into the cerebrospinal fluid induced sickness behaviour with an increase in the production of pro-inflammatory mediators and increased expression of NF-κβ mRNA in the hippocampus. Similar effects were revealed *in vitro* in microglia cells stimulated with dsHMGB1. However, injection of frHMGB1 did not result in the same effects.^34^ In another study, separate injections of dsHMGB1 and frHMGB1 into the cerebral cortex of rats initiated local inflammation characterised by increased IL-1β production and significant blood–brain barrier disruption.^35^ In a study by Morioka et al., astrocytic TLR4 was activated after intrathecal treatment with HMGB1 in mice, which led to increased production of IL-1β and increased neuropathic pain.^36^

Fatigue after cerebral strokes is common. However, the mechanisms involved are unclear. Increasing evidence points to systemic inflammatory responses and sickness behaviour induced by pro-inflammatory cytokines as important factors in post-stroke fatigue.^37^ To date, there are no human studies on the role of HMGB1 in post-stroke fatigue. However, in an experimental animal stroke model, it was observed that the release of HMGB1 and signalling through RAGE contributed to brain injury–induced sickness behaviour. Anti-HMGB1 Ab treatment of the animals reduced sickness behaviour, but it did not improve the infarct volume or the outcome.^38^ These observations strengthen the hypothesis on the role of HMGB1 in fatigue generation.

In Crohn’s disease, intestinal inflammation is believed to result from a complex interplay between environmental factors and a dysregulated innate immune system in a genetically predisposed individual.^39^ Macrophages and monocytes at inflamed intestinal sites are potential sources for the extracellular secretion of DAMPs such as HMGB1, where dsHMGB1 and frHMGB1 may act by binding to TLR4 and RAGE receptors, respectively.^40^

HMGB1 can interact with several pathogen recognition receptors to promote cell migration and cytokine production, as well as act in complex with other immune-stimulating molecules such as IL-1β, LPS and DNA and enhance their pro-inflammatory responses.^41–44^ HMGB1 appears to be involved in the pathophysiology of IBD. McDonnell et al. reported increased blood levels of HMGB1 in patients with Crohn’s disease and ulcerative colitis, but they saw no association with disease activity.^11^ Likewise, they found no association between anti-HMGB1 Abs in blood and disease activity markers (CRP, f-calprotectin and SES-CD). However, a study that measured faecal HMGB1 levels reported a positive correlation with endoscopic disease activity.^45^

A somewhat intriguing finding was that both dsHMGB1 and frHMGB1 were strongly associated with fatigue. We originally hypothesised that dsHMGB1 may have a more central role in fatigue through canonical binding to TLR4 on macrophages and microglia and inducing IL-1β production and release. We found that anti-frHMGB1 Abs were as strongly associated with fatigue severity as were anti-dsHMGB1 Abs. Whether this was due to frHMGB1 signalling through RAGE or oxidation of frHMGB1 by ROS to dsHMGB1 is unclear.^46^,^47^

There are several limitations to this study. First, we did not measure the blood concentrations of HMGB1. Doing so for the redox variants dsHMGB1 and frHMGB1 in particular would have been interesting. It is well known that anti-HMGB1 Abs interfere with conventional ELISAs for HMGB1 measurements,^48^ and currently, no accurate and reliable method is available for measuring total HMGB1 or its redox variants.^49^ Second, we did not measure the levels of IL-1β or other molecules that are known to influence fatigue severity or induce sickness behaviour. Third, we found no difference in the anti-HMGB1 Ab concentrations between healthy subjects and patients. Our findings, after adjusting for non-specific binding by subtracting absorbance from uncoated wells, are opposed to those of Taikashi et al., who reported higher levels in patients with Crohn’s disease.^50^ Whether the fine specificity of anti-HMGB1 Abs that occurs in a disease state implies that these Abs have functional activity as opposed to what is seen in healthy states remains to be clarified. A fourth point that could be discussed is the age difference between patients and healthy subjects. However, in our experience, age does not influence fatigue levels.^51^,^52^ We also find it unlikely that the age difference observed would influence auto-Ab levels significantly. A last point to consider is that the secondary Ab in the assay captures all Ab isoforms. It is possible that measures of specific isoforms could have provided more information about their function and source.

In conclusion, we found that the blood concentration of anti-HMGB1 Abs in patients with Crohn’s disease strongly influences the severity of fatigue. We hypothesise that in acute and chronic inflammatory conditions, anti-HMGB1 Abs down-regulate HMGB1-induced inflammation. This leads to lower production of pro-inflammatory cytokines and lower sickness behaviour and fatigue.
